# Wilms Tumor-1 (*WT1*) rs16754 Polymorphism

**DOI:** 10.4274/tjh.galenos.2018.2018.0407

**Published:** 2019-02-07

**Authors:** Pathum Sookaromdee, Viroj Wiwanitkit

**Affiliations:** 1 TWS Medical Center, Bangkok, Thailand; 2 Dr. DY Patil University, Pune, India

**Keywords:** Wilm’s Tumor, gene, polymorphism

## To the Editor,

The publication “Wilm’s tumor-1 (WT1) rs16754 Polymorphism and Clinical Outcome in Acute Myeloid Leukemia” was very interesting [1]. Ramzi et al. [1] noted that “*WT1* rs16754 polymorphism may be a reliable independent prognostic factor”. In fact, the *WT1* rs16754 polymorphism is proposed as an important prognostic marker for leukemia [2]. The application in the present study might be expected. However, there can also be interference effects of other polymorphisms (such as MDM2 [3] and long non-coding RNA GAS5 polymorphism [4]) that were not investigated that might modify the value of *WT1* rs16754 polymorphism testing. Single polymorphism study might limit its application in clinical usage for prognosis prediction for leukemia patients.

## References

[1] Ramzi M, Moghadam M, Cohan N (2019). Wilm’s tumor-1 (WT1) rs16754 polymorphism and clinical outcome in acute myeloid leukemia. Turk J Hematol.

[2] Petiti J, Rosso V, Lo Iacono M, Calabrese C, Signorino E, Gaidano V, Berger M, Saglio G, Cilloni D (2018). Prognostic significance of the Wilms’ tumor-1 (WT1) rs16754 polymorphism in acute myeloid leukemia. Leuk Res.

[3] He X, Chen P, Yang K, Liu B, Zhang Y, Wang F, Guo Z, Liu X, Lou J, Chen H (2015). Association of MDM2 polymorphism with risk and prognosis of leukemia: a meta-analysis. Acta Haematol.

[4] Yan H, Zhang DY, Li X, Yuan XQ, Yang YL, Zhu KW, Zeng H, Li XL, Cao S, Zhou HH, Zhang W, Chen XP (2017). Long non-coding RNA GAS5 polymorphism predicts a poor prognosis of acute myeloid leukemia in Chinese patients via affecting hematopoietic reconstitution. Leuk Lymphoma.

